# Long term survival of advanced hepatoid adenocarcinoma of lung secondary to idiopathic pulmonary fibrosis: a case report

**DOI:** 10.3389/fonc.2025.1487334

**Published:** 2025-02-04

**Authors:** Xiaoli Wang, Nan Wei, Huizhen Yang, Xiaoyan Wang, Xiaoju Zhang, Qianqian Zhang

**Affiliations:** ^1^ Department of Respiratory and Critical Care Medicine, Zhengzhou University People's Hospital, Henan Provincial People's Hospital, Zhengzhou, Henan, China; ^2^ Henan International Joint Laboratory of Diagnosis and Treatment for Pulmonary Nodules, Zhengzhou University People's Hospital, Henan Provincial People’s Hospital, Zhengzhou, Henan, China; ^3^ Department of Pathology, Zhengzhou University People's Hospital, Henan Provincial People's Hospital, Zhengzhou, Henan, China

**Keywords:** hepatoid adenocarcinoma, lung cancer, idiopathic pulmonary fibrosis, alpha-fetoprotein, immune checkpoint inhibitor

## Abstract

**Background:**

Alpha-fetoprotein (AFP)-producing hepatoid adenocarcinoma of lung (HAL) is a rare type of lung cancer, with its characteristics being not yet fully clarified. We recently encountered a case of HAL combined with idiopathic pulmonary fibrosis (IPF), which has never been reported.

**Case presentation:**

A 66-year-old man consulted our hospital with a chief complaint of cough. Chest computed tomography (CT) revealed multiple nodules measuring from 8mm to 20mm in diameter located in bilateral lung, along with an enlarged left hilar lymph node. CT-guided percutaneous lung biopsy confirmed the diagnosis of AFP-producing primary HAL combined with IPF. Systemic treatment according to guidelines for advanced non-small cell lung cancer resulted in a long-term survival.

**Conclusions:**

This case report documents the first occurrence and prognosis of AFP-producing HAL in a patient with IPF. The long-term survival brought by the diagnosis and treatment model in our case may provide significant prognostic value for this rare condition.

## Background

Hepatoid adenocarcinoma of the lung (HAL) is an extremely rare tumor type, which was first reported as an Alpha-fetoprotein (AFP)-producing lung cancer by Ysunami (1). The pathological features of HAL were fully defined by Ishikura in 1990 (2) and were modified by Haninger in 2014 (3). Since AFP-producing HAL has scarcely been reported, the clinical features and molecular mechanism of this type of lung cancer are still unclear, with no standard treatment regime being recommended. When concomitant idiopathic pulmonary fibrosis (IPF) was diagnosed, the prognosis of lung cancer maybe much poorer (4–6).

Interestingly, we encountered a case of AFP-producing HAL in a patient with IPF, which benefit from the systematic treatment and achieved a long-term survival for this rare type of lung cancer. To the best of our knowledge, this is the first report of AFP-producing HAL in a patient with IPF.

## Case presentation

**Figure 1:**
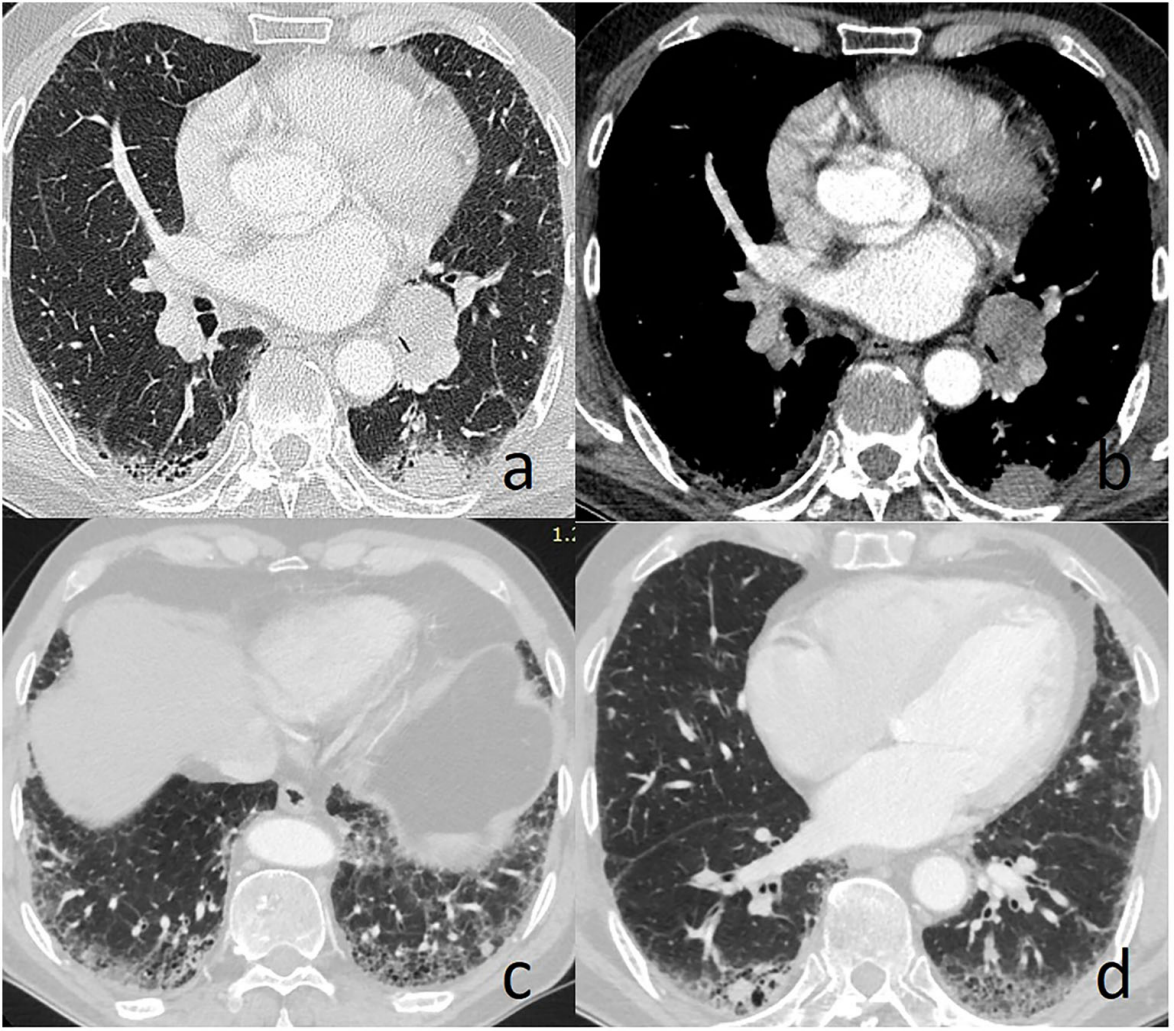
Chest computed tomography reveals left lung nodule and enlarged #11L lymph node **(A, B)**, and multiple lung nodules in the area with obvious fibrotic changes **(C, D)**.

A 66-year-old man was admitted to our hospital with the chief complaint of cough in March 2018. Chest computed tomography (CT) revealed multiple nodules measuring from 8mm to 20mm in diameter located in bilateral lung, along with an enlarged #11L lymph node (**Figures 1A–D**). The patient had been diagnosed of IPF four years prior. Of the tumor markers, most were within the normal range, except for a markedly elevated serum AFP level of 6753ng/ml. A CT-guided percutaneous lung biopsy was performed in the largest pulmonary nodule located in the S6 segment of the left lung.

**Figure 2:**
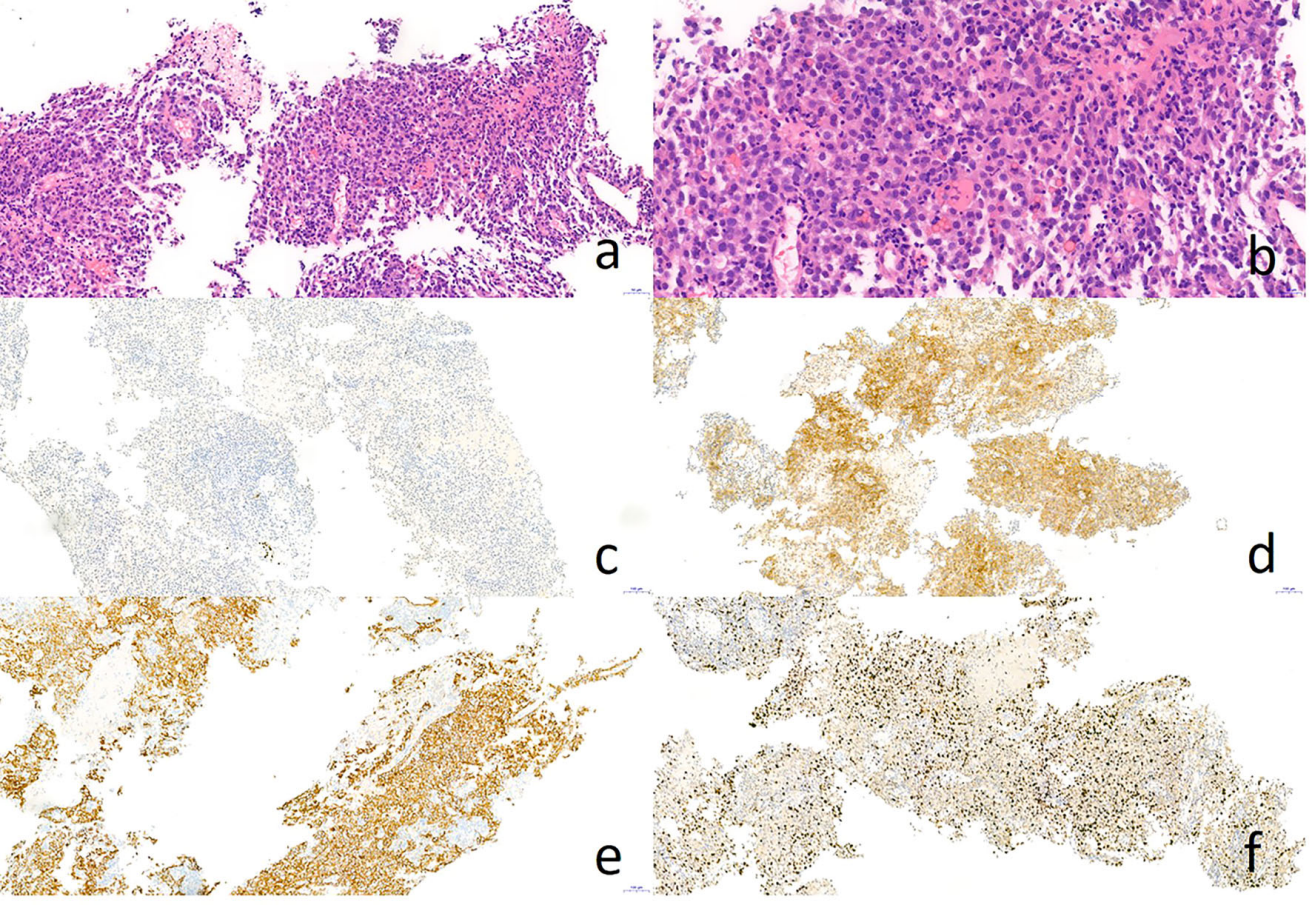
Pathology result of CT-guided percutaneous lung biopsy. Hematoxylin-eosin staining showed poorly differentiated adenocarcinoma like hepatocellular carcinoma **(A, B)**. Immunohistochemical staining showed negative for TTF-1 **(C)** and positive for Hepatocyte **(D)** and CK8/18 **(E)**, as well as Ki-67 40% **(F)**.

Hematoxylin and eosin staining indicated the poorly differentiated cancer cells resembling hepatocellular carcinoma (**Figures 2A, B**). Immunohistochemical staining showed that the tumor cells were negative for TTF-1 (**Figure 2C**), but positive for Hepatocyte (**Figure 2D**) and CK8/18 (**Figure 2E**), with moderate positivity for Ki-67 (40%, **Figure 2F**). Abdominal magnetic resonance imaging (MRI) and CT examination showed no evidence of hepatic or other digestive system tumors. Positron emission tomography/computed tomography (PET-CT) revealed increased standard uptake value (SUV) in the lung nodules (SUV max: 8.1) and in the enlarged #11L lymph node (SUV max: 4.1). No abnormal hypermetabolic lesions were observed in any other organs. Serum biochemical tests did not reveal any evidence of hepatic dysfunction or hepatitis B or C infection. Based on these findings, the patient was diagnosed with AFP-producing primary HAL, clinical stage IV (T4N1M1a). Molecular studies were negative for EGFR, ROS-1, ALK, RET, MET, PIK-3CA, ERBB-2, KRAS, and BRAF mutations.

Subsequently, the patient received six cycles of pemetrexed plus cisplatin chemotherapy, with bevacizumab administered every 21 days concurrently. There was a slight decrease in the AFP serum level (4679 ng/ml), as well as a reduction in the diameter of nodule in left lower lobar. However, due to the side effects, the patient refused further chemotherapy and was instead treated with oral Nintedanib for both lung cancer and IPF. After five months, an increase in the AFP serum level and enlargement of lung nodule and lymph node were noted. Consequently, the patient continued to receive docetaxel as second-line therapy, resulting in partial regression of the pulmonary lesions. Upon subsequent progression, Anlotinib was administrated for 16 months. Since March 2021, the patient has been treated with pembrolizumab, leading to the partial shrinkage of the pulmonary lesion and lymph node. After 2 years of treatment with pembrolizumab, the primary lesion progressed again and pemetrexed plus anlotinib was given for 2 cycles. Due to side effects, chemotherapy was stopped and anlotinib was taken orally intermittently, and the efficacy was evaluated to be stable. The primary lesion developed again 6 months later, and tracheoscopic tissue biopsy was performed, and the pathological results indicated hepatoid adenocarcinoma. The treatment with sintilimab combined with anlotinib lasted for 2 cycles, and the progress of efficacy was evaluated, followed by bronchial artery embolization. The patient is currently being followed up. The patient survived for 75 months following the diagnosis of AFP-producing IPF-HAL.

**Figure 3:**
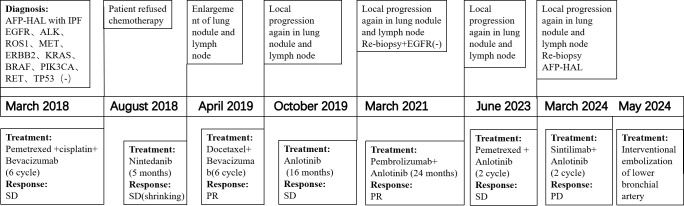
Flow chart of diagnosis and treatment process.

No acute exacerbation of IPF or immune-related injuries associated with chemotherapy or immune checkpoint inhibitors occurred throughout the treatment process. **Figure 3** illustrate the diagnostic and treatment timeline.

## Discussion and conclusions

The incidence of lung cancer in patients with IPF increases with each year following an IPF diagnosis (4–6). Squamous cell carcinoma and adenocarcinoma are the most frequent types of lung cancer in IPF patients, with isolated cases of large cell carcinoma and small cell lung cancer also reported (4). However, to our knowledge, AFP-producing HAL has not been previously reported. Here, we report a case of this rarely type of lung cancer in an IPF patient to raise awareness for clinicians.

Hepatoid adenocarcinoma of the lung is a relatively rare primary malignant tumor of the lung, with an incidence of 0.014/100000 people (7). Clinically, patients with HAL typically presented with nonspecific symptoms. Grossman et al. found that 96% of the tumors occur in men with elevated serum AFP levels and a history of tobacco use. HAL usually presents as a bulky mass in an upper lobe with metastasis and follows an aggressive clinical course (8). HAL closely mimics hepatocellular carcinoma (HCC) and can be misdiagnosed by both pathologists and clinicians, especially when serum AFP level is elevated (9). PET-CT can be used to comprehensively examine patients, aiding in confirm the origin of tumor when the serum AFP levels are elevated (10). Although it is not necessary for the diagnosis of HAL according to recent criteria, serum AFP level is still an important predictive factor in this condition (8, 11).

HAL is an extremely heterogeneous tumor type, and currently, no standard treatment is available. According to the guidelines for diagnosis and treatment of lung cancer, the common treatments for HAL patients include surgical resection, chemotherapy and radiotherapy. Recently, new treatments such as sorafenib, immunotherapy (Anti-PD-L1, Durvalumab), and radiofrequency ablation have been prescribed for HAL (12–16). However, when HAL is combined with IPF, many risk factors must be considered in treatment decisions. Lung status and postoperative complications should be fully evaluated before lung resection. The early and long-term outcomes of surgery in lung cancer patients with IPF are poor due to the high risk of acute exacerbation (AE) of IPF and lung cancer recurrence (17, 18). Radiation and chemotherapy are also known risk factors for AE-IPF (19, 20). With a deeper understanding of IPF and lung cancer, more treatment modalities are being explored (21–23). In our study, surgery and radiation were not performed; instead, long-term survival of 75 months was achieved through sequential chemotherapy (pemetrexed plus cisplatin, Docetaxel), anti-angiogenesis therapy (Bevacizumab, Anlotinib), antifibrotic therapy (Nintedanib), immunotherapy (Pembrolizumab, Sintilimab) and local treatment (Interventional embolization of lower bronchial artery).

In conclusion, this case report is the first to describe the occurrence of AFP-producing HAL in an IPF patient. The disease courses of both IPF and HAL are variable and somewhat unpredictable, potentially altered by the co-occurrence. Currently, there is no consensus on the treatment of patients with both diseases. The long-terms survival achieved in our case may provide prognostic value for this rare condition. Further understanding of the pathogenic overlap between lung cancer and IPF could guide the development of specific diagnostic modalities and targeted treatments for both conditions in the future.

## Data Availability

The original contributions presented in the study are included in the article/supplementary material, further inquiries can be directed to the corresponding author/s.
